# Supplementary material for: CMRSegTools: An open-source software enabling reproducible research in segmentation of acute myocardial infarct in CMR images
Source: PLoS One. 2022 Sep 13;17(9):e0274491. doi: 10.1371/journal.pone.0274491 (PMC9469999; doi:10.1371/journal.pone.0274491)
Supplement: S1 Appendix — (PDF) [file pone.0274491.s001.pdf]

S1. Appendix
Lesion Segmentation Methods in CMRSegTools

William A. Romero R., Magalie Viallon, Joël Spaltenstein,
Lorena Petrusca, Olivier Bernard, Loïc Belle,
Patrick Clarysse, and Pierre Croisille

Contents

1 Introduction **1**

2 Methods **1**
2.1 Manual cut-off (n-SD) . . . 1
2.2 Manual histogram-based segmentation . . . 2
2.3 Full-Width at Half-Maximum (FWHM) . . . 2
2.4 Gaussian Mixture Model . . . 3
2.5 Feature Analysis and Combined Thresholding . . . 3
2.6 Hidden Markov Random Field model with Expectation-Maximisation (HMRF-EM) . . . 4

1 Introduction

This appendix provides a brief description of the computational methods implemented within CMRSegTools for the segmentation of lesions in LGE MR images.

2 Methods

All the methods described below are performed within a subset of the image pixels selected by a prior LV myocardium segmentation method. The LV segmentation returns a non-empty set of pixels inside the endocardial and epicardial contours,

$$LV_{seg} = \{p \mid p \in (endocardium, epicardium)\} \tag{1}$$

where p denotes a pixel at (i, j) image coordinates.

Working on a subset of the image pixels reduces the computational complexity $\mathcal{O}(N)$ (expressed as a function of the image size N) as the algorithm only computes the solutions on the pixels of interest.

2.1 Manual cut-off (n-SD)

This is the most used semi-automatic method based on STRM. Several studies have demonstrated good correlation with pathology. This method requires the definition of a ROI in the remote healthy myocardium (figure 1a). Therefore, the threshold is calculated by

$$T = \mu_{remote} + c\sigma_{remote} \tag{2}$$

where μ is the mean of the signal intensity in the remote healthy myocardium and σ is the standard deviation, c is a positive integer usually between 2 and 10. The subset of the lesion pixels are selected as

$$Lesion_{seg} = \{p \mid T \leq I(p) \wedge p \in LV_{seg}\} \tag{3}$$

where $I(p)$ is the signal intensity of pixel p.

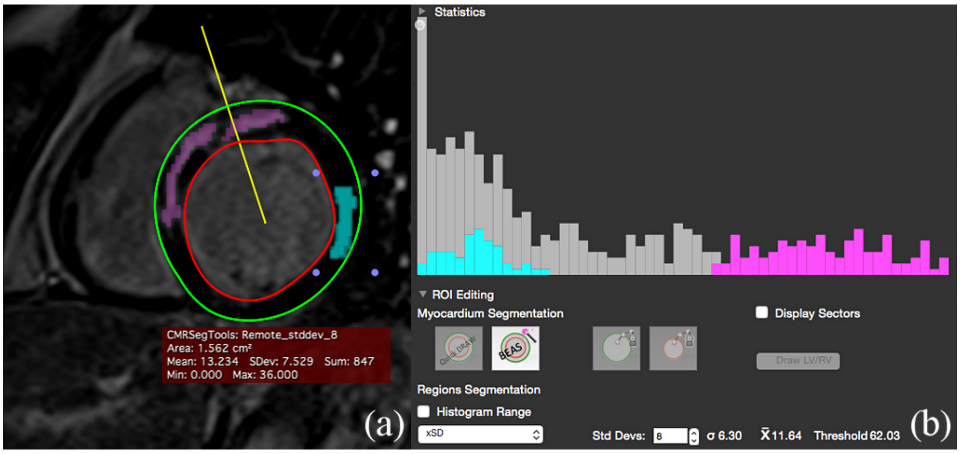

Figure 1: Manual cut-off (n-SD) segmentation. (a) The image viewer displays the Epicardium (green) and Endocardium (red) contours, the LV/RV junction landmark (yellow), the lesion segmentation (magenta) and the remote healthy myocardium (turquoise); (b) The CMRSegTools GUI displays the histogram highlighting the bins from the remote ROI (turquoise) and lesion segmentation (magenta).

2.2 Manual histogram-based segmentation

All the pixels which have an intensity value within the range $\{a, b\}$ are categorised as lesion

$$Lesion_{seg} = \{p \mid a \leq I(p) < b\} \tag{4}$$

where a and b are the range limits set by the user on the GUI with the lower and upper cursors on the histogram or using the Histogram Range text boxes (2b).

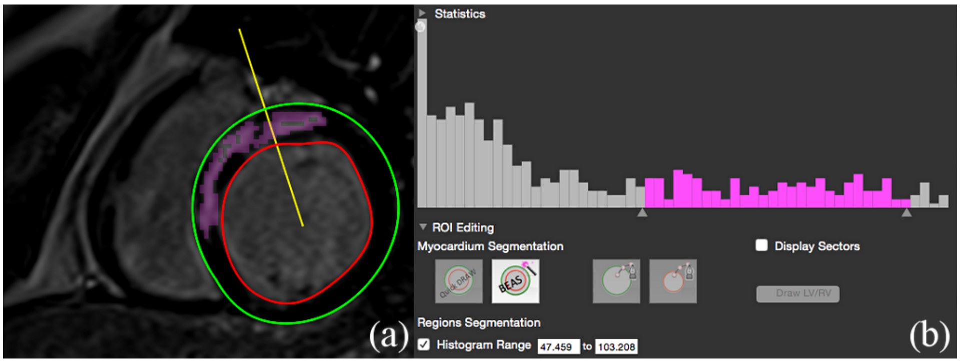

Figure 2: Manual histogram-based segmentation. (a) The image viewer displays the Epicardium (green) and Endocardium (red) contours, the LV/RV junction landmark (yellow) and the lesion segmentation (magenta); (b) The CMRSegTools GUI displays the histogram highlighting the bins between the lower and upper threshold manually defined on the histogram or in the Histogram Range text boxes.

2.3 Full-Width at Half-Maximum (FWHM)

In this automatic method, the threshold is calculated as

$$T = \frac{I_{max}}{2} \tag{5}$$

where I_{max} is the maximum signal intensity within the myocardium (LV_{seg}). CMRSegtools includes the following options:

- FWHM Max. The threshold is calculated from the maximum SI within the myocardium of the k-th slice in K slices.

$$T(k) = \frac{I_{max}(k)}{2} \tag{6}$$

- FWHM Region. This method requires the definition of a ROI in the healthy myocardium. The threshold is calculated as

$$T = \frac{I_{max} + \mu_{remote}}{2} \tag{7}$$

where μ is the mean of the signal intensity in the remote myocardium.

- FWHM Region3D. The method is a generalisation of the FWHM Region. The threshold is calculated with the maximum SI in the k-th slice and the mean SI of a unique remote ROI defined in slice k_{remote}.

$$T(k) = \frac{I_{max}(k) + \mu_{remote}(k_{remote})}{2} \tag{8}$$

2.4 Gaussian Mixture Model

This approach models the histogram of intensity values x (i.e. SI) in the myocardium as

$$f_R\left(x;\mu,\sigma^2\right) = w_R f_R\left(x;m,\sigma_R^2\right) + w_G f_G\left(x;\mu,\sigma_G^2\right) \tag{9}$$

where w_R corresponds to the relative proportion of healthy tissue and w_G to the lesion respectively (Rician-Gaussian mixture model [1]). The probability density function (PDF) of the Gauss distribution is

$$f_G\left(x;\mu,\sigma_G^2\right) = \frac{1}{\sqrt{2\pi\sigma_G^2}} \times e^{-\frac{(x-\mu)^2}{\sigma_G^2}} \tag{10}$$

where μ and σ^2 are the mean and variance of the distribution respectively, parameters to be identified by the algorithm. The PDF of the Rice distribution is

$$f_R\left(x;m,\sigma_R^2\right) = \frac{x}{\sigma_R^2} \times e^{-\frac{x^2+m^2}{2\sigma_R^2}} \times I_0\left(\frac{xm}{\sigma_R^2}\right) \tag{11}$$

where m is a parameter and $I_0(z)$ is the modified Bessel function of the first kind with order zero [2]. The algorithm relies on an Expectation–Maximisation (EM) method to estimate the parameters of the mixture model.

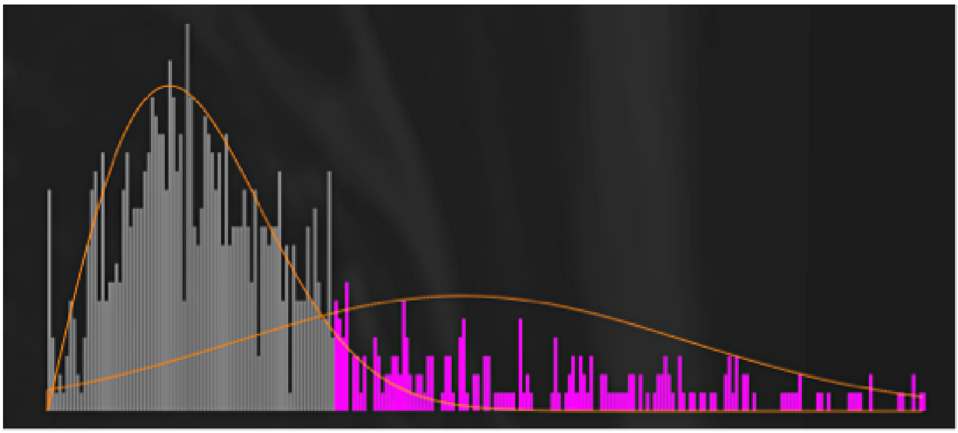

Figure 3: Visualisation on CMRSegTools of the distributions calculated by the Gaussian Mixture Model method on the histogram.

2.5 Feature Analysis and Combined Thresholding

The feature analysis and combined thresholding (FACT) algorithm proposed by Hsu et al. in [3] is a workflow composed of the following steps:

1. *Histogram clustering.* The GMM method (Section 2.4) is applied in order to find the mean μ and the standard deviation σ of the healthy myocardium and define a binary image by applying

$$g(k,p) = \begin{cases} 0 & \text{if } I(k,p) < T \\ 1 & \text{otherwise} \end{cases} \tag{12}$$

where $I(k,p)$ is the signal intensity of pixel p at slice k, and $T = \mu + c\sigma$.

2. *Region-Based Feature Analysis.* On each slice k, isolated 2D regions are grouped to 3D volumes (stack of 2D slices) by using a neighbour connectivity criteria providing a 3D binary image (volume) with the voxels of interest. In order to remove false positive voxels, the following filters are performed:

   - Volume filter removes regions smaller than a minimum setting based on the myocardial mass density.
   - Distance filter removes any region more than 2 mm away from the endocardial border.
   - Intensity filter removes voxels about 50% darker than the average intensity of a 3D region.

3. *FWHM Thresholding.* In order to remove voxels from partial volume tissues, a threshold defined as $T = \frac{I_{max}+\mu_{remote}}{2}$ is applied. Steps 2 and 3 can be repeated in order to reduce false positives voxels/regions.

4. *Detection of the MVO regions.* A morphology closing filter is used to identify small holes surrounded by bright infarcted voxels. These small holes are then labeled as no-reflow regions.

2.6 Hidden Markov Random Field model with Expectation-Maximisation (HMRF-EM)

This method has been developed based on the algorithm proposed by Zhang et al. [4] in which the objective is to define the corresponding labels $\mathbf{x} = \{x_1, x_2, x_3, ..., x_N\}$ for the pixels in an image $\mathbf{y} = \{y_1, y_2, y_3, ..., y_N\}$ where y_i is the SI of the i-th pixel. In the case of CMR, the set of labels $\mathcal{L}$ in the myocardium (LV_{seg}) is defined as $\mathcal{L} = \{l_1, l_2, l_3, ..., l_M\}$ (namely $\mathcal{L} = \{normal, \ lesion\}$). Within a Maximum likelihood framework, the method relies on the solution to the following optimisation problem:

$$\mathbf{x} = \arg\max_{x} \{P(\mathbf{y} \mid \mathbf{x}, \mathbf{\Theta})P(\mathbf{x})\} \tag{13}$$

where $\mathbf{\Theta}$ is the set of parameters (mean and standard deviation) of the PDF that defines each label; which is written formally as $\mathbf{\Theta} = \{\theta_l = (\mu_l, \sigma_l) \mid l \in \mathcal{L}\}$. The estimation of these set of parameters is made using the EM algorithm.

The HMRF-EM workflow in CMRSegTools includes the following steps [5]:

1. *Cartesian to polar transformation of the LV_{seg} region.* A polar space is better adapted to the myocardium morphology. In the polar space neighbour operations are more effective as pixels in the same circumference (cartesian space) are aligned in the same line (polar space). Figure 4 shows the output of the Cartesian to polar transformation.

2. *Initial classification.* An initial label map and the corresponding parameters (mean and standard deviation) are computed by finding the SI which is most likely to be lesion. This initial classification uses a threshold defined as $T = \frac{4 \times I_{max}}{5}$ with I_{max} given by 4. This step provides the initial parameter set $\mathbf{\Theta}^{(0)}$ for the label map $\mathbf{x}^{(0)}$.

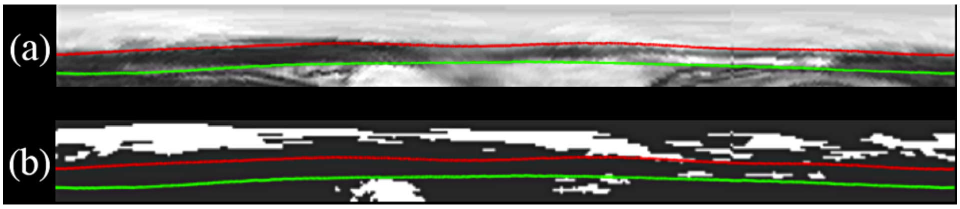

Figure 4: Output of the Cartesian to polar transformation of the LV_{seg} region and initial classification including Epicardium (green) and Endocardium (red) contours. (a) Polar image of the LV_{seg} region in which angles are along the horizontal axis and radius along the vertical axis; (b) Output label map $\mathbf{x}^{(0)}$ of the initial classification.

3. *EM algorithm.* The t-th iteration of the process, the *Expectation* step determines the conditional expectation of the parameters

$$Q\left(\mathbf{\Theta} \mid \mathbf{\Theta}^{(t)}\right) = E\left[ln\left(P(\mathbf{x}, \mathbf{y} \mid \mathbf{\Theta})\right) \mid \mathbf{y}, \mathbf{\Theta}^{(t)}\right] \tag{14}$$

$$= \sum_{x} P(\mathbf{x} \mid \mathbf{y}, \mathbf{\Theta}^{(t)})\, ln\left(P(\mathbf{x}, \mathbf{y} \mid \mathbf{\Theta})\right) \tag{15}$$

and the *Maximisation* step calculates the next estimation of parameters

$$Q\left(\mathbf{\Theta} \mid \mathbf{\Theta}^{(t+1)}\right) = \arg\max_{\theta} \left\{ Q\left(\mathbf{\Theta} \mid \mathbf{\Theta}^{(t)}\right) \right\} \tag{16}$$

Maximum a posteriori (MAP) estimation is performed for calculating the labels based on the current set of parameters $\mathbf{\Theta}^{(t)}$

$$\mathbf{x}^{(t)} = \arg\max_{x} \left\{ P(\mathbf{y} \mid \mathbf{x}, \mathbf{\Theta}^{(t)}) P(\mathbf{x}) \right\} \tag{17}$$

$$\mathbf{x}^{(t)} = \arg\min_{x} \left\{ U(\mathbf{y} \mid \mathbf{x}, \mathbf{\Theta}^{(t)}) + U(\mathbf{x}) \right\} \tag{18}$$

where $U(\mathbf{y} \mid \mathbf{x}, \mathbf{\Theta}^{(t)})$ is the likelihood energy distribution. The EM process continues until the energy is stable or a maximum number of iterations is reached (Figure 5).

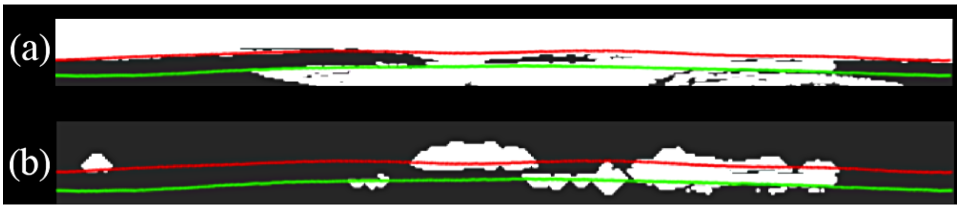

Figure 5: Output of the EM process including Epicardium (green) and Endocardium (red) contours. (a) Label map $\mathbf{x}^{(t)}$ at the t-th iteration; (b) label map $\mathbf{x}$ at the end of the EM iterative process.

4. *Post-processing and identification of MVO regions.* The LV_{seg} region is transformed from polar to Cartesian. A connected component analysis is applied to remove small clusters (i.e. less than 0.1 g) or isolated clusters too distant from the endocardium (distance above 1.5 mm), removing false positives. As the MVO region can be completely inside the lesion region or at the edge of myocardial boundaries, connected components which size is over 50% of the myocardium size are removed. External bordering pixels that are not labeled as lesion are removed if they are above a defined threshold (set to 50%).

Figure 6 illustrates the HMRF-EM workflow.

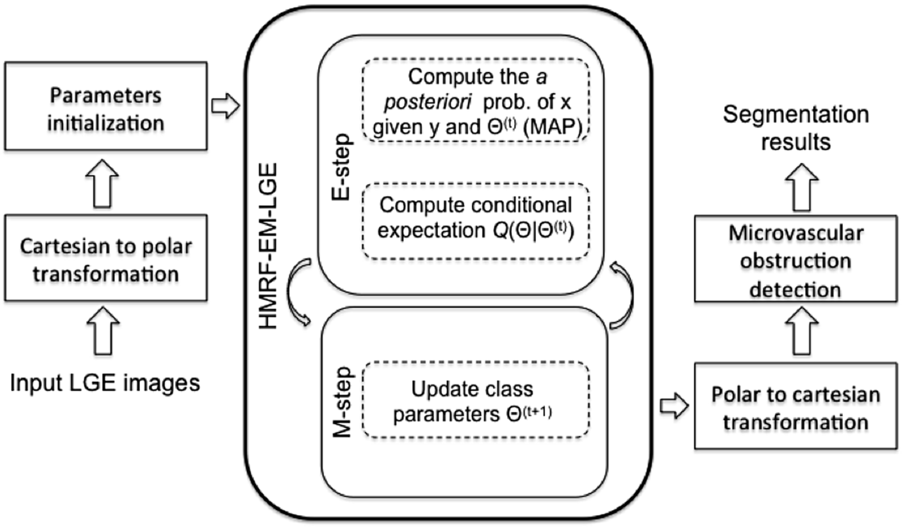

Figure 6: HMRF-EM workflow [5].

References

[1] O. Friman, A. Hennemuth, and H. Peitgen, “A rician-gaussian mixture model for segmenting delayed enhancement mri images,” in *Proc. 16th Sci. Meeting Int. Soc. Magn. Reson. Med.(ISMRM’08)*, 2008, p. 1040.

[2] S. O. Rice, “Mathematical analysis of random noise,” *The Bell System Technical Journal*, vol. 23, no. 3, pp. 282–332, 1944.

[3] L.-Y. Hsu, A. Natanzon, P. Kellman, G. A. Hirsch, A. H. Aletras, and A. E. Arai, “Quantitative myocardial infarction on delayed enhancement mri. part i: Animal validation of an automated feature analysis and combined thresholding infarct sizing algorithm,” *Journal of Magnetic Resonance Imaging: An Official Journal of the International Society for Magnetic Resonance in Medicine*, vol. 23, no. 3, pp. 298–308, 2006.

[4] Y. Zhang, M. Brady, and S. Smith, “Segmentation of brain mr images through a hidden markov random field model and the expectation-maximization algorithm,” *IEEE Transactions on Medical Imaging*, vol. 20, no. 1, pp. 45–57, 2001. DOI: 10.1109/42.906424.

[5] M. Viallon, J. Spaltenstein, C. de Bourguignon, *et al.*, “Automated quantification of myocardial infarction using a hidden markov random field model and the em algorithm,” in *International Conference on Functional Imaging and Modeling of the Heart*, Springer, 2015, pp. 256–264.
