# Supplementary material for: CMRSegTools: An open-source software enabling reproducible research in segmentation of acute myocardial infarct in CMR images
Source: PLoS One. 2022 Sep 13;17(9):e0274491. doi: 10.1371/journal.pone.0274491 (PMC9469999; doi:10.1371/journal.pone.0274491)
Supplement: Attachment — Submitted filename: Response to Reviewers.pdf [file pone.0274491.s002.pdf]

12th June 2022

Dear Editor,

We thank the reviewers for their comments on the manuscript “CMRSegTools: an open-source software enabling reproducible research in segmentation of acute myocardial infarct in CMR images”. We have edited the manuscript to address their concerns. Please find our response and revised manuscript enclosed.

We have also reformatted the manuscript in line with the requirements for submission of revised manuscripts. We believe that the manuscript is now suitable for publication in PLOS One.

Thank you for your consideration.

Yours sincerely,

William A. Romero R.

William A. Romero R.
PhD. Candidate
University of Lyon
Lyon, France

Editor comments

1. Please ensure that your manuscript meets PLOS ONE's style requirements, including those for file naming.

Thank you. We have followed the style according to the style format of PLOS ONE. We used the Template for PLoS-Version 3.5 March 2018 Latex version.

2. Thank you for stating the following financial disclosure:

"The funders had no role in study design, data collection and analysis, decision to publish, or preparation of the manuscript."

We have included the financial disclosure.

This work has been performed within the framework of LABEX PRIMES (ANR-11-LABX-0063) of Université de Lyon, within the program "Investissements d'Avenir" (ANR-11-IDEX-0007) and France Life Imaging (ANR-11-INBS-0006) operated by the French National Research Agency (ANR). The funders had no role in study design, data collection and analysis, decision to publish, or preparation of the manuscript.

3. We note that you have stated that you will provide repository information for your data at acceptance. Should your manuscript be accepted for publication, we will hold it until you provide the relevant accession numbers or DOIs necessary to access your data. If you wish to make changes to your Data Availability statement, please describe these changes in your cover letter.

The source code is available at

https://github.com/OpenCardiacMRISoftware/CMRSegTools

and we included the link within the paper. In the same direction, we included the links to the data used for validating the software:

http://humanheart-project.creatis.insa-lyon.fr/

Response to reviewers

Manuscript PONE-D-22-07768 entitled "CMRSegTools: an open-source software enabling reproducible research in segmentation of acute myocardial infarct in CMR images"

We thank the Editor for the opportunity to respond to the reviewers' comments. Please find our response and revised manuscript enclosed.

Reviewer #1

1. The paper presents an open-source software for segmentation of CMR-LGE images. Quantitative LGE analysis is not strictly required by current CMR guidelines in the clinical setting (J Schulz-Menger JCMR 2020 10.1186/s12968-020-00610-6), but it is needed in clinical and translational research. A large number of LGE segmentation algorithms was proposed (Wu Front. Physiol 2021 | 10.3389/fphys.2021.709230), few of them established in the clinical practice.

We thank the reviewer for the observation and references. We have included the latter reference in our bibliographical revision and discussion.

The following paragraph has been added to the Introduction:

For instance, a recent publication of Wu et al. [5] presents a general review of cardiac scar segmentation methods (including methods for non-LGE images). This review reports quantitative results across publications. The conclusion states the need of a public benchmarking of the methods as a fairer review of their performances. This is a challenge that requires tools for open collaboration in a growing culture focused on advancing methods by reproducible science [6, 7]. The neuroimaging research community is a clear example of this research and development methodology [8, 9].

The following sentence has been also added to the Introduction:

These approaches require annotated data sets which are generated and assessed by standard methods (i.e. signal threshold versus reference mean, full-width at half-maximum, etc.) [4, 5, 19, 20]

The following reference has been added:

5. Wu, Yinzhe, Zeyu Tang, Binghuan Li, David Firmin, and Guang Yang. "Recent Advances in Fibrosis and Scar Segmentation From Cardiac MRI: A State-of-the-Art Review and Future Perspectives." Frontiers in Physiology 12 (2021).

2. About available software, several certified, commercial tools for CMR-LGE analysis are available at high cost from several companies. Some groups developed software for internal use but in my knowledge the only available free tools is the Segment software from Medvisio(https://medviso.com/cmr/) running by a free available Matlab VM on about all SO platforms. Segment is free usable if proper referencing is assured (https://medviso.com/how-to-refer/) but source code is not available, so the software cannot be adapted to user needs.

Hence, the introduction of on open-source software is important and may represent an important improvement in the field. I tried the demo version in Horos and it seems to work well. Basic video tutorials are provided, maybe a manual would be also useful.

Collaboration terms required for the use in scientific publication of the software (likely the only possible use as the software is of course not certified) are proper referencing as usually requested in the field and mandatory co-authorship of developers. The last request is not usual in open-source software sharing as co-authorship is usually applied id a specific developing work is done for a particular project. I guess that the GNU/CeCILL license doesn't allow the developer to add further limitations with respect to the license itself. Is also not clear how this "co-authorship license" should apply if thanks to the open-source approach the software will be improved or modified by other developers external to the starting team. The "co-authorship license" constraint, if maintained, should be declared in the paper as well as it represents a significant limitation.

We thank the reviewer for testing our application software and pointing out the potential limitations of the use of CMRSegTools under the current "co-authorship licence".

Before releasing CMRSegTools as an open-source software, we provided a binary version of the plug-in as well as scientific expertise in applied computing in Cardiac MRI and software customisation to the particular needs of the users. This makes us active collaborators in the research projects using the application software. In this context, the co-authorship agreement has been working among research teams using CMRSegTools.

Now our interest is to join efforts through an open collaboration. Releasing the source code of CMRSegTools together with this publication is our first step. The GNU/CeCILL is the French free software license, compatible with the GNU GPL, the license grants access to the source code and the rights to copy, modify and redistribute the code.

We have also included Segment CMR (https://medviso.com/cmr) and Cass MR Solutions (https://www.piemedicalimaging.com/product/mr-solutions) in our software comparison.

The sentence below has been added to the Discussion section:

In this direction, CMRSegTools source code has been released under CeCILL licence (French free software license compatible with GPL) that grants users the right to copy, modify, and distribute new versions of the plug-in.

3. Validation of the software is mostly anecdotic as single cases as presented without quantitative assessment. Is strongly advisable to perform a comparison with a validated software or manual analysis by an expert user on a reference data set including a reasonable number of cases, as MICCAI challenges or similar.

We thank the reviewer for this observation. The software has already been tested before this open-source release. All implemented methods have been evaluated in the publications cited in appendix S1.

The following paragraph has been added to the CMRSegTools plug-in section:

BEAS was originally developed for automatic segmentation of 3D cine-loop echocardiographic images to provide an efficient, fast and accurate solution for quantification of the main left ventricular volumetric indices used in clinical routine. In addition, the method was adapted for 3D+time CMR data sets (acquired by a cine steady state free precession sequence, SSFP) and benchmarked against the data sets available from the MICCAI 2009 Cardiac MR Left Ventricle Segmentation Challenge [33]. The benchmark demonstrated the technique to be robust, efficient and fast in terms of accuracy and computational load, which makes the BEAS algorithm suitable for a clinical practice [32].

And in the Discussion section:

To validate the correspondence between the mathematical model (S1 appendix) and the code implementation, the correctness of the computational methods has been verified with synthetic data.

An extended assessment or benchmarking of infarct segmentation methods is beyond the scope of this paper. The goal is to provide a computer-based tool which includes well-known lesion segmentation methods (S1 appendix) in order to empower research scientists to reproduce results and assess comparatively new methods. In this direction, CMRSegTools source code has been released under CeCILL licence (French free software license compatible with GPL) that grants users the right to copy, modify, and distribute new versions of the plug-in.

4. Table 1 compares general-use software (that of course does not match R1/R2 requirements) with CMR specific ones that of course match both as required by SCMR guidelines. Research/Commercial Segment software should be inserted in the table together with other commercial software as Pie CAAS (https://www.piemedicalimaging.com/product/mr-solutions/tissue-characterisation). The compatibility with different OS should be indicated, as one limitation of the proposed software is the requirement of MacOS system.

We thank the reviewer for this recommendation. We have included Segment CMR and CAAS MR Solutions to the software comparison. In addition, we

have included a column "Target Platform" in Table 1. We have added comments on the limitation to deploy CMRSegTools on a different operative system.

The Table 1 has been completed as:

Table 1. Overview of available post-processing tools. Requirement 1 (R1): myocardial segmentation, requirement 2 (R2): statistical analysis and visualisation following the AHA 17-segment model.

Application Software	Description	R1	R2	Target Platform	Licence
3D Slicer	Subject-specific biomedical image processing and visualisation.	No	No	Linux, Windows, macOS.	Open-source, BSD-style.
ImageJ	Multi-purpose scientific image processing and visualisation.	No	No	Linux, Windows, macOS.	Public-Domain, BSD-2.
MeVisLab	Workflow-based fast-prototyping	No	No	Linux, Windows, macOS.	Proprietary, Freeware.
Medis Suite MR	Cardiac MR image analysis	Yes	Yes	Windows	Commercial
Circle CVI42	Cardiac MR image analysis	Yes	Yes	Windows, macOS	Commercial
Segment CMR	Cardiac MR image analysis	Yes	Yes	MATLAB. Linux, Windows, macOS.	Segment open licence agreement.
Caas MR Solutions	Cardiac MR image analysis	Yes	Yes	Windows	Commercial
CMRSegTools	Cardiac MR image analysis	Yes	Yes	macOS	CeCILL

And a paragraph added to the Discussion section:

CMRSegTools is therefore a contribution to enable reproducible research by assembling the most used methods for infarct segmentation in a suitable workflow executed in a single and widely available environment: Osirix/Horos DICOM viewers. This platform was chosen because it provides an Application Programming Interface (API) that grants access to the data management, user interaction and visualisation functionalities. It was also chosen because it has an open-source licence. However, OsiriX/Horos is a macOS-only software, which limits its deployment on platforms based on a different operative system.

Reviewer #2

This study presents a open-source software for myocardial infarction segmentation in CMR images. There are some concerns:

1. It is better to brief introduce the licenses shown in Table 1 for clarifying the difference in different software.

We thank the reviewer for this recommendation. We have added a brief overview of licences in the Introduction:

In general, commercial software is a product designed to be marketed under a utilisation licence. The user must pay for a binary version which must be used as described by the licence. There is no access to the source code of the commercial product (closed-source). Freeware software, is free of charge but closed-source. In open-source software, the user has access to the source code and the different licences (i.e. BSD, GNU GPL, etc.) provide the rights to copy, modify, redistribute the code, as well as build and release binary versions with commercial purposes. [11]. Open-source software promotes open collaboration and research communities have been attracted to this innovation methodology [12].

The following sentence has been added to the Discussion section:

In this direction, CMRSegTools source code has been released under the CeCILL licence (French free software license compatible with GPL) that grants users the right to copy, modify, and distribute new versions of the plug-in.

2. Is there any comparison of BEAS with other myocardial contour segmentation ? If not, please explain the reason.

We thank the reviewer for this important remark.

In CMRSegTools plug-in section, the paragraph below has been added:

BEAS was originally developed for automatic segmentation of 3D cine-loop echocardiographic images to provide an efficient, fast and accurate solution for quantification of the main left ventricular volumetric indices used in clinical routine. In addition, the method was adapted for 3D+time CMR data sets (acquired by a cine steady state free precession sequence, SSFP) and benchmarked against the data sets available from the MICCAI 2009 Cardiac MR Left Ventricle Segmentation Challenge [33]. The benchmark demonstrated the technique to be robust, efficient and fast in terms of accuracy and computational load, which makes the BEAS algorithm suitable for a clinical practice [32].

3. It is better to cite more studies in cardiac and myocardial infarction segmentation. 1) Direct delineation of myocardial infarction without contrast agents using a joint motion feature learning architecture, Medical Image Analysis; 2) Multi-level semantic adaptation for few-shot segmentation on cardiac image sequences, Medical Image Analysis.

We thank the reviewer for the observation and references. We have included Xu C., et al. 2018 and Guo S. et al., 2021 in our bibliographical revision and discussion.

In the Discussion section we added,

At present, there are no automated algorithms that can reliably distinguish the bright LV cavity from the bright endocardial border of the infarct. This challenge is being addressed by new segmentation methods (including non-LGE images) based on ML [5,20, 47, 48].

In the References, we added

47. Xu C, Xu L, Gao Z, Zhao S, Zhang H, Zhang Y, et al. Direct delineation of myocardial infarction without contrast agents using a joint motion feature learning architecture. Medical image analysis. 2018;50:82-94.

48. Guo S, Xu L, Feng C, Xiong H, Gao Z, Zhang H. Multi-level semantic adaptation for few-shot segmentation on cardiac image sequences. Medical Image Analysis. 2021;73:102170.

4. Please briefly discuss the limitation of this software

In the Discussion section, the following comment has been added:

CMRSegTools is therefore a contribution to enable reproducible research by assembling the most used methods for infarct segmentation in a suitable workflow executed in a single and widely available environment: Osirix/Horos DICOM viewers. This platform was chosen because it provides an Application Programming Interface (API) that grants access to the data management, user interaction and visualisation functionalities. It was also chosen because it has an open-source licence. However, OsiriX/Horos is a macOS-only software, which limits its deployment on platforms based on a different operative system.

5. A lot of grammatical errors.

We have thoroughly reviewed and improved the grammar of the article.

6. Please present a figure to show the modules and their connections in this software.

We thank the reviewer for this recommendation. We added the diagram below presenting the main components and package dependencies in the CMRSegTools plug-in section:

Figure 1 shows the main components of the plug-in and its integration within the OsiriX/Horos environment.

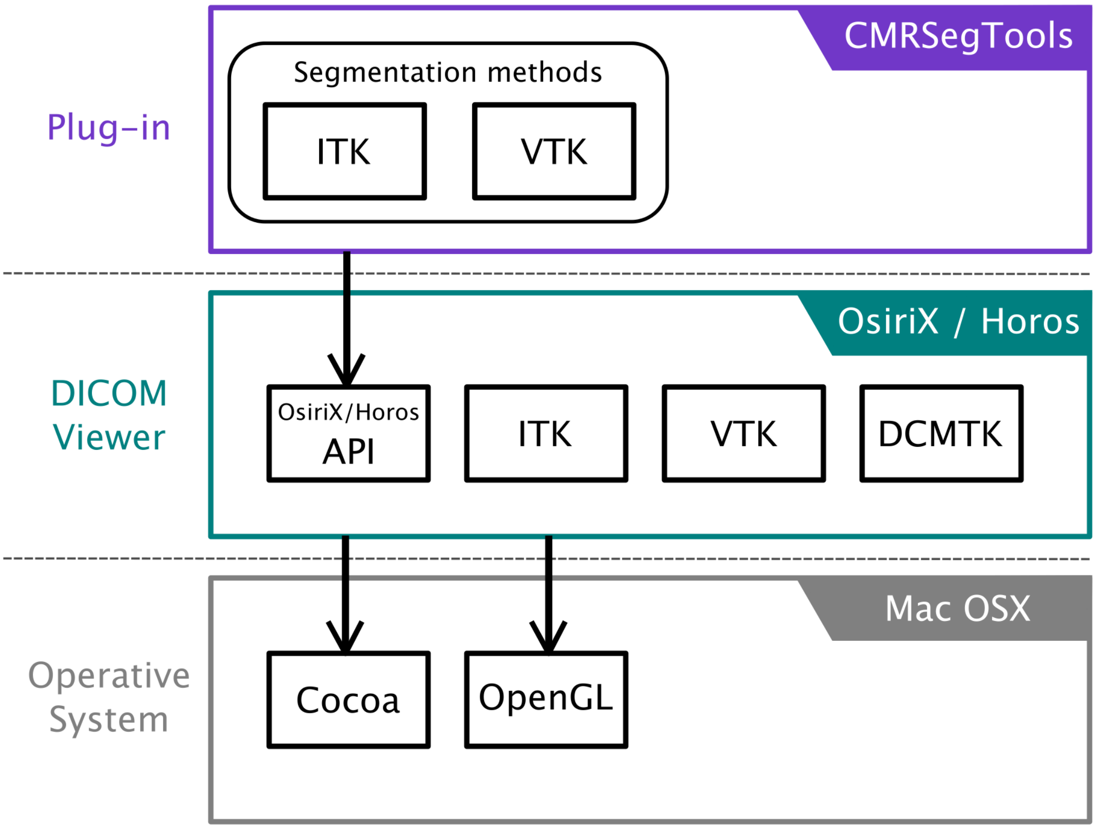

Figure 1. CMRSegTools architecture diagram. The plug-in has access to the DICOM viewer functionalities through the Application Programming Interface (API) exposed by OsiriX/Horos. List of the frameworks and toolkits in each layer: ITK, Insight Toolkit; VTK, Visualisation Toolkit; DCMTK, DICOM Toolkit; Cocoa, Mac OSX Native API; OpenGL, Computer Graphics API.
