# Supplementary material for: CMRSegTools: An open-source software enabling reproducible research in segmentation of acute myocardial infarct in CMR images
Source: PLoS One. 2022 Sep 13;17(9):e0274491. doi: 10.1371/journal.pone.0274491 (PMC9469999; doi:10.1371/journal.pone.0274491)
Supplement: Attachment — Submitted filename: Response to Reviewers.pdf [file pone.0274491.s003.pdf]

19th August 2022

Dear Editors,

We thank the reviewers for their comments on the manuscript “CMRSegTools: an open-source software enabling reproducible research in segmentation of acute myocardial infarct in CMR images”. We have edited the manuscript to address their concerns. Please find our response and revised manuscript enclosed.

We have also reformatted the manuscript in line with the requirements for submission of revised manuscripts. We believe that the manuscript is now suitable for publication in PLOS One.

Thank you for your consideration.

Yours sincerely,

William A. Romero R.
PhD. Candidate
University of Lyon
Lyon, France

Manuscript PONE-D-22-07768 entitled "CMRSegTools: an open-source software enabling reproducible research in segmentation of acute myocardial infarct in CMR images"

We thank the Editor for the opportunity to respond to the reviewers' comments. Please find our response and revised manuscript enclosed.

Response to reviewers

Reviewer #1: Authors have addressed my requests. I have some very minor comments:

1. radiology expert experience should be quantified (e.g. > 10 years)

We thank the reviewer for this important remark.

The following paragraph has been edited in the section Radiology expert segmentation vs automated segmentation methods:

An example of the manual segmentation made by an expert radiologist (P.C. with 20 years of experience in cardiovascular imaging) against each one of the methods within CMRSegTools on a patient with MVO is presented in figures 7 and 8.

2. Reference 25 should be updated (pages 32(7):4340-4351)

We thank the reviewer for the observation. The reference has been updated and following the PLOS One reference style the reference 25 is written as follows:

25. Zeilinger MG, Kunze KP, Munoz C, Neji R, Schmidt M, Croisille P, et al. Non-rigid motion-corrected free-breathing 3D myocardial Dixon LGE imaging in a clinical setting. European Radiology. 2022;32. doi:10.1007/s00330-022-08560-6.
